# Dispersal of *Mycobacterium tuberculosis* Driven by Historical European Trade in the South Pacific

**DOI:** 10.3389/fmicb.2019.02778

**Published:** 2019-12-04

**Authors:** Claire V. Mulholland, Abigail C. Shockey, Htin L. Aung, Ray T. Cursons, Ronan F. O’Toole, Sanjay S. Gautam, Daniela Brites, Sebastien Gagneux, Sally A. Roberts, Noel Karalus, Gregory M. Cook, Caitlin S. Pepperell, Vickery L. Arcus

**Affiliations:** ^1^School of Science, University of Waikato, Hamilton, New Zealand; ^2^Maurice Wilkins Centre for Molecular Biodiscovery, The University of Auckland, Auckland, New Zealand; ^3^Department of Medical Microbiology and Immunology, School of Medicine and Public Health, University of Wisconsin–Madison, Madison, WI, United States; ^4^Department of Microbiology and Immunology, School of Biomedical Sciences, University of Otago, Dunedin, New Zealand; ^5^School of Medicine, University of Tasmania, Hobart, TAS, Australia; ^6^School of Molecular Sciences, La Trobe University, Melbourne, VIC, Australia; ^7^Swiss Tropical and Public Health Institute, Basel, Switzerland; ^8^University of Basel, Basel, Switzerland; ^9^LabPLUS, Auckland City Hospital, Auckland, New Zealand; ^10^Waikato Hospital, Hamilton, New Zealand; ^11^Department of Medicine, Division of Infectious Diseases, School of Medicine and Public Health, University of Wisconsin–Madison, Madison, WI, United States

**Keywords:** tuberculosis, pathogen, phylodynamics, phylogeography, indigenous people, Pacific, New Zealand

## Abstract

*Mycobacterium tuberculosis* (*Mtb*) is a globally distributed bacterial pathogen whose population structure has largely been shaped by the activities of its obligate human host. Oceania was the last major global region to be reached by Europeans and is the last region for which the dispersal and evolution of *Mtb* remains largely unexplored. Here, we investigated the evolutionary history of the Euro-American L4.4 sublineage and its dispersal to the South Pacific. Using a phylodynamics approach and a dataset of 236 global *Mtb* L4.4 genomes we have traced the origins and dispersal of L4.4 strains to New Zealand. These strains are predominantly found in indigenous Māori and Pacific people and we identify a clade of European, likely French, origin that is prevalent in indigenous populations in both New Zealand and Canada. Molecular dating suggests the expansion of European trade networks in the early 19th century drove the dispersal of this clade to the South Pacific. We also identify historical and social factors within the region that have contributed to the local spread and expansion of these strains, including recent Pacific migrations to New Zealand and the rapid urbanization of Māori in the 20th century. Our results offer new insight into the expansion and dispersal of *Mtb* in the South Pacific and provide a striking example of the role of historical European migrations in the global dispersal of *Mtb*.

## Introduction

Tuberculosis (TB) is caused by the bacterial pathogen *Mycobacterium tuberculosis* (*Mtb*) and other members of the *Mtb* complex (MTBC). The MTBC comprises seven human adapted lineages, which show strong phylogeographic structure and vary in the extent of their global distribution (Hirsh et al., 2004; Hershberg et al., 2008; Gagneux, 2018). The most widely globally dispersed MTBC lineage is lineage 4 (L4), also known as the “Euro-American” lineage (Gagneux et al., 2006; O’Neill et al., 2019). Spatial and temporal patterns of L4 dispersal suggest that it was spread through European colonial migrations to Africa and the Americas (Hirsh et al., 2004; Gagneux et al., 2006; Hershberg et al., 2008; Pepperell et al., 2011; Brynildsrud et al., 2018; O’Neill et al., 2019). Oceania was the last major region to be reached by Europeans and includes the > 1000 islands of Polynesia scattered across the central and southern Pacific Ocean. Little is known about the origins and dispersal of *Mtb* in this region. It is commonly assumed that *Mtb* was introduced to Polynesia with the arrival of European sailors and settlers, however, TB-like lesions in skeletons predating European arrival challenge this view (Buckley et al., 2010).

New Zealand is the largest country in Polynesia and is home to the local indigenous Māori people and the largest diaspora of communities of Pacific peoples globally. This provides a unique setting for the investigation of *Mtb* dispersal and transmission in this region. *Mtb* genotypes in New Zealand Europeans, Māori and Pacific people in New Zealand are dominated by L4 strains (Yen et al., 2013), consistent with introduction of present-day strains by Europeans. Māori and Pacific People in New Zealand are disproportionately affected by TB and collectively account for ∼70% of New Zealand born TB cases (ESR, 2018). Around three-quarters of *Mtb* isolates from Māori and Pacific People have shared molecular typing patterns indicative of recent transmission. The largest *Mtb* cluster in New Zealand identified by related 24-loci MIRU-VNTR typing patterns is known as the “Rangipo” cluster. This strain predominantly occurs in Māori, accounting for around one-quarter of Māori TB cases (J Sherwood, ESR, personal communication). Two other large clusters known as the “Southern Cross” and “Otara” clusters predominately occur in Pacific people. Globally, indigenous people are generally found to have higher rates of TB than non-indigenous people (Tollefson et al., 2013). Understanding how the pathogen was dispersed and is maintained among indigenous populations is important for designing improved strategies for TB control in these often disproportionally affected populations.

The most common L4 sublineage in New Zealand is L4.4, which accounts for 43% of New Zealand born L4 cases (Stucki et al., 2016). Here, we show that the Rangipo and Otara clusters both belong to the L4.4 sublineage. We sought to examine the global evolutionary history of L4.4 and trace the dispersal of these strains to New Zealand. For this purpose, we analyzed a genomic dataset of 236 *Mtb* L4.4 isolates from 19 different countries including 23 recent and newly sequenced genomes from New Zealand clinical isolates belonging to the Rangipo and Otara clusters. We find the New Zealand strains belong to a L4.4.1.1 “S-type” sublineage clade that includes the DS6^Quebec^ lineage, which was dispersed to Western Canadian First Nations by French-Canadian fur traders in the 18th–19th centuries (Pepperell et al., 2011). Our results suggest migration of this clade to the South Pacific was driven by the expansion of European trade networks in the 19th century, with the whaling trade serving as a likely dispersal route to indigenous Pacific populations. Our findings show the dispersal and expansion of these strains in the Pacific is related to historical and social drivers of TB transmission and provide new insights into the dispersal of this globally successful human pathogen.

## Results

### Phylogeny of the New Zealand *Mtb* Strains

We first performed single nucleotide polymorphism (SNP) based lineage assignment on 34 New Zealand *Mtb* Rangipo, Otara and Southern Cross strain genomes, these included sequences from previous work (Gautam et al., 2017; Mulholland et al., 2017) and new sequencing data. This assigned Rangipo and Otara isolates to the L4.4.1.1 (S-type) sublineage and Southern Cross to L4.3.3 (LAM). A total of 23 New Zealand L4.4 genomes (16 Rangipo, 7 Otara) spanning a 22-year period (1991–2013) met mapping quality thresholds and were included in downstream analyses (Supplementary Table S1 and Supplementary Figure S1). A whole-genome SNP phylogeny of these strains shows the Rangipo and Otara strains form two well-differentiated monophyletic clades with differing phylogenetic structures (Supplementary Figure S2). The Rangipo cluster is characterized by short terminal branches and low genetic diversity (pairwise SNPs 0–12, median 4) suggesting recent clonal expansion and temporally short transmission chains, consistent with its association with local outbreaks. Conversely, Otara isolates have long terminal branches and higher genetic diversity (pairwise SNPs 1–102, median 91). This is consistent with predominant reactivation disease due to a locally endemic strain rather than a recent transmission cluster as previously thought based on MIRU-VNTR typing.

### Global Phylogeny of the L4.4 Sublineage

**FIGURE 1:**
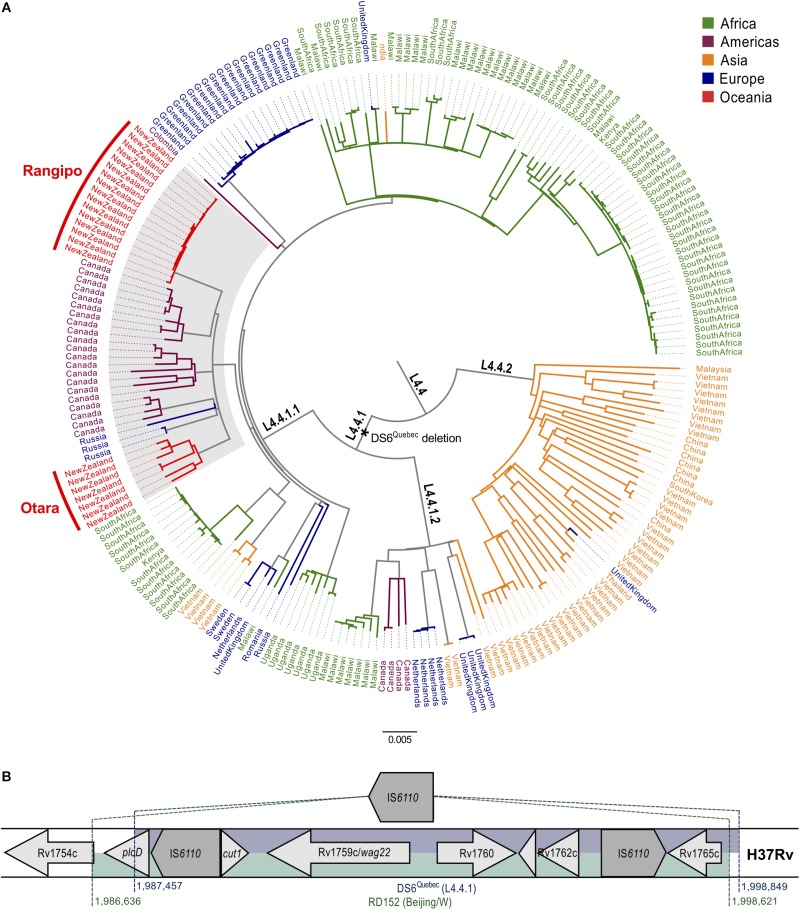
Global phylogeny of the *Mycobacterium tuberculosis* complex L4.4 sublineage and genomic structure and phylogenetic placement of the DS6^Quebec^ deletion. **(A)** Whole genome SNP maximum likelihood phylogeny of L4.4 comprised of 236 isolates from 19 different countries, including 23 isolates from the New Zealand Rangipo and Otara clusters. Scale bar indicates substitutions per polymorphic site. A black asterisk indicates the DS6^Quebec^ deletion and the CS clade is highlighted in gray. Branches and tip labels are colored by geographic region and lineages labeled according to the nomenclature of Coll et al. (2014). A black circle indicates the node position of the most recent common ancestor of L4.4 (rooted to H37Rv, not shown). **(B)** Schematic of the DS6^Quebec^ deletion. Genomic regions in H37Rv that are deleted by the DS6^Quebec^ deletion and the RD152 deletion in the Beijing/W lineage are shown.

To investigate the origins and dispersal of the New Zealand L4.4 strains, we compiled a dataset comprising our 23 New Zealand Rangipo and Otara strain genomes and 213 L4.4 genomes from 18 different countries representing all five major global regions (Supplementary Figure S3 and Supplementary Table S2). WGS reads were mapped to the H37Rv reference genome and repetitive genomic regions were removed prior to alignment. High quality variant sites were extracted producing a 9024 bp SNP alignment used to infer a global L4.4 maximum likelihood phylogeny (Figure 1A and Supplementary Figure S4). This shows L4.4 comprises three well-differentiated sublineages, L4.4.1.1, L4.4.1.2 and L4.4.2 (pairwise *F*_*ST*_ values between lineages 0.50–0.57), consistent with the Coll classification system (Coll et al., 2014).

L4.4 has previously been observed at high proportions in parts of Asia and Africa (Stucki et al., 2016). Consistent with this observation, a large proportion of isolates in our dataset come from these regions (Supplementary Figure S3). Our results reveal differing global distributions and population structures of the L4.4 sublineages. We find L4.4.2 is essentially restricted to Eastern Asia consistent with *in situ* growth and diversification of this sublineage. Conversely, both L4.4.1.1 and L4.4.1.2 are relatively well distributed globally, indicative of high rates of migration and efficient dispersal.

Within L4.4.1.1, we identified a clade comprised predominantly of isolates from New Zealand and Canada. The Canadian isolates belong to the DS6^Quebec^ lineage, which is endemic in French Canadians in Quebec and Western Aboriginal Canadian populations, and is characterized by the presence of the DS6^Quebec^ deletion (Nguyen et al., 2004; Pepperell et al., 2011). We named the clade encompassing the New Zealand strains and Canadian DS6^Quebec^ lineage isolates the “CS” clade (colonial S-type) for its association with European colonial activities in Canada (Pepperell et al., 2011) and the Pacific (shown in this work). Examination of mapped sequencing reads found that both of the New Zealand clusters carry the DS6^Quebec^ deletion and the presence of the deletion in the Rangipo and Otara strains was further confirmed by PCR and Sanger sequencing. The Rangipo and Otara clusters are not monophyletic within the CS clade, consistent with at least two separate introductions into New Zealand.

### Phylogenetic Placement of the DS6^Quebec^ Deletion

The DS6^Quebec^ deletion removes an approximately 11.4 kb region truncating or removing the genes from Rv1755c/*plcD* to Rv1765c (between positions 1987457 to 1998849 in H37Rv) (Nguyen et al., 2004; Figure 1B). Examination of mapped reads found that all L4.4.1.1 and L4.4.1.2, but not L4.4.2 genomes, harbored the DS6^Quebec^ deletion, showing that this is a characteristic deletion of L4.4.1. One L4.4.1.1 genome had an earlier start to the deletion (position 1987142) indicating a subsequent small deletion event. This same region is also removed by the similar but evolutionarily independent ∼12 kb RD152 deletion (positions 1986636 to 1998621) in L2/Beijing strains (Figure 1B; Tsolaki et al., 2005). The genomic region affected by RD152 and DS6^Quebec^ is highly variable and is associated with frequent insertion of IS*6110* elements (Ho et al., 2000), suggesting homologous recombination between adjacent IS*6110* elements as the likely mechanism responsible for these similar but evolutionarily distinct deletion events.

### Temporal Evolution of the L4.4.1.1 Sublineage and the CS Clade

The polytomy at the root of the CS clade and the polyphyletic nature of the New Zealand and Canadian isolates implies dispersal of several closely related strains from a common origin. It is likely that the DS6^Quebec^ lineage was introduced to Canada from France (Pepperell et al., 2011), suggesting a similar European origin for the New Zealand strains. French whalers had a notable presence in New Zealand and Polynesia during the South Pacific whaling era (1790–1860) (Haines, 2010) and the arrival of whalers and other traders in the region is associated with the introduction of new diseases, including TB (Lange, 1984; Chappell, 1997). We hypothesized the CS clade may have been introduced to the Pacific via this route. To further explore this hypothesis, the temporal evolution and dispersal of the L4.4.1.1 sublineage was investigated by Bayesian evolutionary analysis using BEAST2 (Bouckaert et al., 2014) with an alignment of 3161 variable nucleotide positions from 117 L4.4.1.1 genomes, which included all New Zealand isolates and global L4.4.1.1 isolates with known year of isolation at the time of analysis (Supplementary Table S3). Both root-to-tip regression (*R*^2^ = 0.229) and date randomization tests detected sufficient temporal signal in the data set for calibration of the molecular clock by tip-dating (Supplementary Figure S5).

The L4.4.1.1 phylogeny, mutation rate and node ages were inferred using strict and relaxed molecular clocks with different coalescent demographic models. Nucleotide substitution rate was modeled using the general time reversible (GTR) model. All models produced similar rate and date estimates (median 6.15 × 10^–8^–6.64 × 10^–8^ substitutions per site per year (sub/site/yr); widest 95% highest posterior density (HPD) intervals over all models, 4.23 × 10^–8^–9.08 × 10^–8^) (Table 1). Model comparison using path sampling determined that the strict clock with the Bayesian skyline demographic model provided the best fit to the data (Supplementary Table S5). Under this model we estimated a substitution rate of 6.28 × 10^–8^ sub/site/yr (95% HPD, 4.54 × 10^–8^–8.10 × 10^–8^), resulting in a time to most recent common ancestor (TMRCA) estimate of 1492 for L4.4.1.1 (95% HPD, 1325–1629). Our substitution rate estimate is similar to the results from other studies using contemporary L4 and mixed lineage MTBC genomes, all of which produced median rate estimates of ∼7 × 10^–8^–1 × 10^–7^ sub/site/yr (Ford et al., 2013; Pepperell et al., 2013; Roetzer et al., 2013; Walker et al., 2013; Eldholm et al., 2015).

**TABLE 1:** *Mycobacterium tuberculosis* complex L4.4.1.1 sublineage substitution rate and time to most recent common ancestor (TMRCA) estimates.

**Clock model**	**Demographic model**	**Substitution rate (×10^–8^)**	**L4.4.1.1 TMRCA**	**CS clade TMRCA**	**Rangipo TMRCA**	**Otara TMRCA**
Strict	Constant	6.63 (4.34–9.06)	1513 (1301–1673)	1671 (1529–1777)	1978 (1965–1987)	1827 (1744–1886)
Strict	Exponential	6.63 (4.37–9.08)	1513 (1299–1672)	1672 (1530–1781)	1978 (1965–1986)	1827 (1745–1887)
**Strict**	**Skyline**	**6.28 (4.54–8.10)**	**1492 (1325–1629)**	**1652 (1535–1741)**	**1980 (1969–1988)**	**1813 (1746–1868)**
UCLD	Constant	6.49 (4.23–8.93)	1500 (1277–1667)	1665 (1518–1774)	1977 (1964–1986)	1824 (1741–1887)
UCLD	Exponential	6.64 (4.37–8.98)	1511 (1294–1667)	1673 (1528–1772)	1977 (1965–1986)	1828 (1748–1888)
UCLD	Skyline	6.15 (4.39–7.98)	1480 (1300–1624)	1645 (1524–1743)	1980 (1968–1988)	1809 (1739–1867)

*Median values are reported and 95% HPD interval shown in brackets. Substitution rate is in substitutions per site per year. The best fitting model and estimates reported in the text are shown in bold. The GTR model of substitution was used for all analyses.*

**FIGURE 2:**
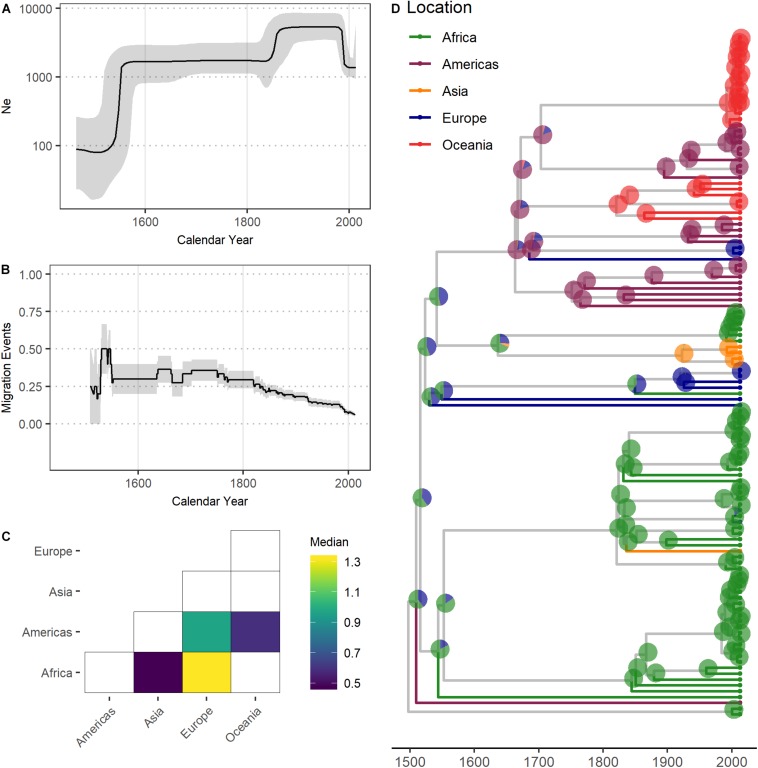
Demographic analysis of the *Mycobacterium tuberculosis* complex L4.4.1.1 sublineage. **(A)** Effective population size (N_e_) through time of L4.4.1.1. Median N_e_ and 95% highest posterior density pictured as black line and gray shading, respectively. *X*-axis in calendar years. **(B)** Migration events through time of L4.4.1.1. Black line depicts the rate of migration through time, calculated as the sum of migration events occurring across every year of the phylogeny divided by the total number of branches during each year of the phylogeny. Gray shading depicts the rates inferred after the addition or subtraction of a single migration event. *X*-axis in calendar years. **(C)** Migration matrices of L4.4.1.1. Heatmap of pairwise relative migration rates between UN regions. Only relative rates with Bayes factor > 5 shown. **(D)** MCC phylogeny of L4.4.1.1. Tips and terminal branches colored according to UN region of isolation. Pie charts on nodes colored according to geographic state probabilities. *X*-axis in calendar years.

The Bayesian skyline plot suggests the L4.4.1.1 sublineage underwent a rapid population expansion following its emergence, and corresponding migration analyses show a spike in migration at this time (Figures 2A,B). This was followed by a period where the population size remained consistent until another period of population growth in the 19th century, during which time migration tapers off. Our phylogeographic reconstruction is indicative of connectivity between Africa and Europe and shows a pattern of dispersal from Europe to Canada and Oceania (Figures 2C,D). These patterns of connectivity and the dispersal of L4.4.1.1 through Africa and Europe are consistent with previous reconstructions of the migratory history of L4 (O’Neill et al., 2019).

**FIGURE 3:**
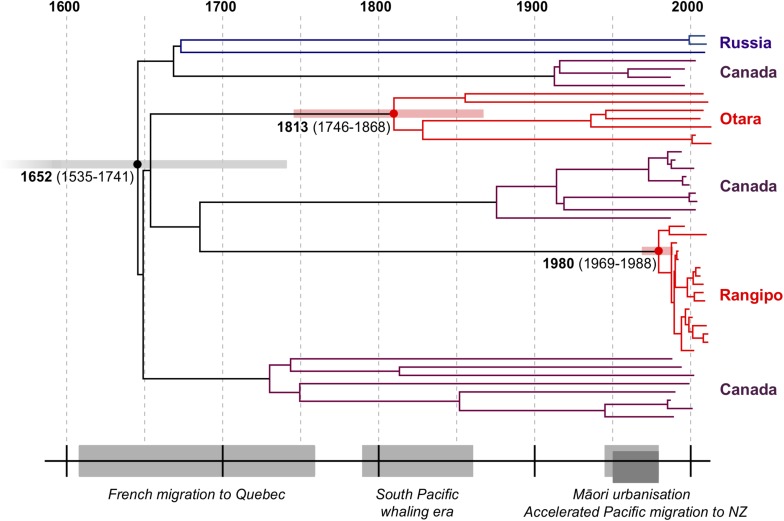
Dated Bayesian phylogeny of the CS clade and historical timeline. Median TMRCA estimates and 95% HPD intervals are shown for the CS, Rangipo, and Otara clades. Events shown include the French migration to Quebec (1608–1760), the South Pacific whaling era (1790–1860), the rapid urbanization of Māori in the 20th century (1945–1980), and the surge in Pacific migration into New Zealand in the 1950s–1970s.

Our estimated TMRCA of the CS clade is 1652 (95% HPD, 1535–1741) and the TMRCA of Rangipo and the closest Canadian clade was 1691 (95% HPD, 1588–1776) (Table 1 and Supplementary Figure S6). This is coincident with the French migration to Quebec between 1608–1760 (Charbonneau et al., 1993), and is thus consistent with a European, likely French, origin of the CS clade (Figure 3). The TMRCA estimate for the Otara strain is 1813 (95% HPD, 1746–1868), which coincides with arrival of European whalers and other traders, including sealers, bêche-de-mer and sandalwood traders, to the Pacific region the early 19th century (Campbell, 2011; Fischer, 2013). Our TMRCA estimate of the Rangipo strain is 1980 (95% HPD, 1969–1988), indicating this strain is either a relatively recent introduction or clonal expansion from a previously introduced, unsampled, CS strain.

## Discussion

Using a phylodynamics inference from WGS data, we have performed a global characterization of the L4.4 sublineage and identify a L4.4.1.1 sublineage clade that is common in indigenous populations in Canada and Polynesia. Molecular dating estimated the MRCA for this clade, which we term the “CS” clade, to have existed in the mid-17th century, which is coincident with the French migration to Quebec and thus consistent with a French origin as previously reported for the DS6^Quebec^ lineage (Pepperell et al., 2011; Figure 3). Our results indicate multiple migrations of closely related CS strains out of Europe to Canada and to the South Pacific, providing a striking example of the role of European colonial and trade migrations in driving the global spread of L4.

The early 19th century TMRCA estimate for the Otara strain fits with an introduction to the Pacific by French/European whalers or other traders (Figure 3). To date, the Otara strain has predominantly been identified in Pacific people living in New Zealand. Considering the recent history of migration to New Zealand from the Pacific Islands, our results suggest this strain was initially dispersed to the Pacific Islands from Europe and subsequently migrated to New Zealand. Although limited data are available on early migration from the Pacific, only very small numbers of Pacific people began to settle in New Zealand in the 1800s and in 1916 there were only 151 Pacific Island people in New Zealand (United Nations and Economic Social Commission for Asia and the Pacific, 1985). The Pacific population in New Zealand began to slowly increase in the first half of the 20th century until an upsurge in migration in the 1950s–1970s (Dunsford et al., 2011), and by 1976 the Pacific population in New Zealand had increased to 61,354 (United Nations and Economic Social Commission for Asia and the Pacific, 1985). The TMRCA of the Otara strain is therefore consistent with initial dispersal to the Pacific Islands by Europeans in the early 1800s. Long internal branches in the phylogeny stretching back to the 19th century suggest multiple subsequent introductions to New Zealand that may have accompanied more recent Pacific migrations.

Unlike New Zealand, which began to receive large influxes of European, predominantly British and Irish, migrants following British annexation in 1840 (United Nations and Economic Social Commission for Asia and the Pacific, 1985), other Polynesian islands did not experience the same *en masse* arrival of European emigrants, lending further support to this being a trade-associated introduction. The whaling trade was also the only French economic activity of any scale in the South Pacific during the early 19th century (Maclellan, 1998), the most significant years of which were 1832–1846 (Foucrier, 2005). As with the Canadian fur trade, commercial success of the whaling industry depended on establishing productive social and economic relationships with the local people. During the whaling era, large numbers of Pacific peoples relocated from villages to harbor settlements for trade and employment opportunities, and Pacific men were often recruited as crew on whaling ships accounting for up to one-fifth of European whaling crews (Chappell, 1997; Fischer, 2013). Intermarriage also played a central role in industry establishment and success in both the Canadian fur trade and New Zealand whaling (Stevens and Wanhalla, 2017). Such interactions would have established strong social ties conducive for the dispersal of *Mtb.* Accordingly, contact with European trade vessels and ports have been implicated in the introduction of TB and other infectious diseases into the South Pacific (Lange, 1984; Chappell, 1997).

The L4.4.1.1 sublineage defined by SNP genotyping corresponds to the S lineage, also known as the “S-type,” classified by spoligotyping (Brudey et al., 2006; Coll et al., 2014). Molecular typing has shown that the S lineage also has a notable presence in French Polynesia, accounting for over one-third of *Mtb* isolates in Tahiti (10/27, 37%) (Osman et al., 2017). Tahiti was made a French protectorate in 1842 and a colony in 1880, and was an important commerce hub provisioning European whaling and trade vessels in the early 19th century. Although no WGS data were available for inclusion in phylogenetic analyses, we speculate that this lineage may have been introduced to Tahiti via the same historical migrations that introduced the New Zealand CS strains to Polynesia. In addition to CS strains in Canada and New Zealand, the CS clade also contains isolates from Russia. Unlike New Zealand and Canada where CS strains occur at relatively high frequencies in indigenous populations, L4.4 is rare in Russia (Casali et al., 2014; Stucki et al., 2016). Historically, Western Europe and Russia have been culturally and politically more connected and trade between them dates back to ancient times (Öhberg, 1955). Russia was also engaged the colonial fur trade (Rich, 1955), providing possible avenues for dispersal of CS strains.

The Rangipo cluster has been responsible for numerous TB outbreaks for over the last 30 years (De Zoysa et al., 2001; McElnay et al., 2004; Colangeli et al., 2014) and is an important source of TB in Māori. Unlike the older endemic Otara strain, our results indicate that the Rangipo cluster arose from a relatively recent clonal expansion, due to either a more recent introduction or emergence from a previously introduced CS strain. Between 1840–1843 the majority of French whaling voyages included New Zealand (70/81, 86.4%) (Foucrier, 2005) and French whaling provides a conceivable route for historical introduction of CS strains into New Zealand from France. Alternatively, Rangipo may be a more recent introduction. The TMRCA follows a period of mass migration to New Zealand from the Pacific Islands in the 1950s–1970s offering another plausible route. Although it is evident that the Rangipo cluster has ultimately emerged from a strain of European origin, more in-depth sampling of L4.4.1.1 isolates from both New Zealand and the Pacific may provide a clearer picture of the route this strain took from Europe to New Zealand and will shed additional light on the dispersal of this sublineage in the South Pacific region.

The Rangipo strain was named for its association with a large TB outbreak in the late-1990s involving cases who had spent time in the Rangipo prison (De Zoysa et al., 2001). Prior to this, health professionals were aware of clusters of infection caused by this strain first appearing in the early 1990s (N. Karalus, personal communication). Our TMRCA estimate for the Rangipo cluster predates this outbreak, although its introduction into the prison environment has presumably helped contribute to its further spread. The TMRCA of the Rangipo strain coincides with major demographic changes in the Māori population that occurred in the mid-20th century. Māori TB mortality rates declined sharply in the mid-1900s (MacLean, 1964), which presumably would have imposed a bottleneck on the *Mtb* population. Along with falling TB rates, between 1945–1980 Māori also experienced one of the fastest rates of urbanization of any population in the world (Pool, 1991). This was accompanied by significant social and environmental changes including overcrowded housing and increased prison incarceration rates, both of which are TB risk factors (Clark et al., 2002; Baussano et al., 2010). These host social and environmental changes are intricately tied to the colonial history of New Zealand. Globally, indigenous people generally have a higher prevalence of “proximate determinants” of TB such as smoking and food insecurity (Cormier et al., 2019). These may be substantial contributors to the high burden of TB in these communities and their prevalence is often linked to upstream social factors (Cormier et al., 2019). The temporal association between the emergence of the Rangipo cluster and the urbanization of Māori suggests that human social phenomena are important contributors to the expansion and dispersal of *Mtb* in indigenous populations. A similar pattern is also observed in Canada, whereby *Mtb* population expansion in indigenous Canadian populations occurred concomitant with major environmental and social changes affecting host populations (Pepperell et al., 2011).

Both Rangipo and Otara are Māori place names and Otara is a city that is home to large populations of Pacific people, associating these names with Māori and Pacific people more generally. Our results show that these strains are a product of European contact and colonization and highlight the pejorative naming of these strains with Māori names. Naming diseases by place of origin stigmatizes the associated population and the name “Rangipo” also further perpetuates the stigma attached to the disease by associating it with prison and criminality. Stigma increases the emotional suffering of TB patients and has implications for TB control efforts, for example by affecting health-seeking behaviors and adherence to treatment (Craig et al., 2017). The findings of this work point to the appropriateness of renaming these clusters to refrain from further stigmatizing communities where TB is present and perpetuating stigma associated with the disease, and further work will seek to formally rename these clusters in consultation with Māori.

Recently, Brynildsrud et al. (2018) reconstructed the migratory history of the L4 sublineage, including isolates from Europe, Africa, the Americas and Southeast Asia. Global dispersal of L4 was found to be dominated by historical migrations out of Europe and dispersal of L4 to Africa and the Americas occurred concomitant with European colonial migrations (Brynildsrud et al., 2018). We observe the same scenario with the introduction of L4.4 to the South Pacific and the CS clade provides a striking example of the role of European expansion in the global dispersal of *Mtb*. Our analyses reveal the migration of several closely related CS strains out of Europe in the 17th–19th centuries to remote and unconnected populations driven by European colonial migrations and expanding trade networks. In a separate study by O’Neill et al. (2019), the evolutionary history of L4 was found to be characterized by rapid diffusion and high rates of migration, with range expansion contributing to the growth of this lineage. Consistent with this, our results suggest efficient dispersal of L4.4 and a more extensive demographic analysis of the L4.4.1.1 sublineage revealed increased population growth concurrent with a spike in migration in the 16th century following emergence of this lineage. This timing is coincident with the European age of exploration, providing a plausible factor that may have contributed to the growth and dispersal of this sublineage. A similar pattern of increased population growth and migration during this era has also been detected for L4 as a whole (O’Neill et al., 2019). We detect L4.4.1.1 population growth in the 19th century that could be attributable to various colonial activities around this time involving countries represented in our sample; the French-Canadian fur trade (1710–1870) (Innis, 1999), the South Pacific whaling trade (1790–1860) (Haines, 2010), and the rapid occupation and colonization of much of the African continent during the New Imperialism period (1876–1912) (Pakenham, 2015). The later population decline in the late 20th century coincides with the dramatic decline in TB incidence in the developed world over the last century.

The phylogeography of bacterial pathogens can provide valuable insights into the migratory history of their human hosts. Most notably, *Helicobacter pylori* has been identified as reliable marker to deduce human population movements, providing valuable insights into ancient human migrations in the Pacific and globally (Falush et al., 2003; Moodley et al., 2009). In this study, we identify multiple migrations of several closely related *Mtb* strains to geographically distant and unconnected indigenous populations driven by European colonial and trade expansion in the 17–19th centuries. The presence of the CS clade in indigenous Pacific populations provides a potential marker of these historical migrations and reasserts the role of European migrations in the global dispersal of L4 *Mtb*. Our results highlight the power of phylodynamic methods and the utilization of public WGS data repositories to trace recent migrations of *Mtb* in high resolution at both the global and local scale, uncovering human movements and social changes that have contributed to the dispersal and success of *Mtb* in indigenous Pacific populations.

## Materials and Methods

### Genomic Data

#### New Zealand L4.4 Genomes

We have recently sequenced 18, seven and five *Mtb* isolates from the New Zealand Rangipo, Otara and Southern Cross clusters, respectively, on the Illumina MiSeq platform (Mac Aogáin et al., 2016; Gautam et al., 2017; Mulholland et al., 2017). An additional four Rangipo genomes were sequenced for this study using paired-end 250-bp reads on the Illumina MiSeq platform using the Nextera^TM^ XT DNA Library Preparation Kit (Illumina Inc., CA, United States) as previously described (Aung et al., 2016). These four strains have been previously sequenced on the SOLiD platform (Colangeli et al., 2014) and were re-sequenced here on Illumina for inclusion in this study. Sequencing data were submitted to the National Centre for Biotechnology Information (NCBI) Nucleotide Archive (PRJNA356104).

MTBC lineage was determined with KvarQ (v0.12.3a1) (Steiner et al., 2014) using the coll14 testsuite (Coll et al., 2014). This classified Rangipo and Otara isolates as belonging to the L4.4.1.1 sublineage. Southern Cross isolates belonged to L4.3.3 and were excluded from further analysis. Nineteen previously sequenced Rangipo and Otara genomes and all four new Rangipo genomes met mapping quality thresholds for inclusion in this study (depth of coverage > 25X, > 75% reads mapped to the reference genome, < 10% missing calls in alignments), resulting in a dataset of 23 New Zealand L4.4 strains included in further analyses (Supplementary Table S1). The Rangipo strain isolates (*n* = 16) span a 20-year collection period (1991–2011) and include isolates from different geographical locations. MIRU-VNTR data was available for seven of these isolates, all of which share the 24-loci MIRU-VNTR profile that distinguishes the Rangipo cluster (233325153324-341444223362). The remaining isolates had been identified as Rangipo strain based on IS*6110*-RFLP typing and/or contact tracing information. Otara isolates (*n* = 7) spanned an 10-year collection period (2003–2013) and represent four variant MIRU-VNTR typing profiles distinguishable by a single locus.

#### Global L4.4 Genomes

Global L4.4 genomes included 23 newly sequenced genomes from Canada and 190 publicly available genomes from published studies (Bryant et al., 2013a, b; Clark et al., 2013; Walker et al., 2013; Zhang et al., 2013; Casali et al., 2014; Guerra-Assunção et al., 2015a, b; Bjorn-Mortensen et al., 2016; Brynildsrud et al., 2018; Holt et al., 2018) and Broad Institute sequencing initiatives (broadinstitute.org) (Supplementary Table S2). Publicly available genomes were assembled from a list of 13,067 L4 genomes (Stucki et al., 2016) from which those belonging to L4.4 were selected after being identified with KvarQ (*n* = 401) (Supplementary Figure S1). Country and year of isolation were obtained from the NCBI BioSample database. Genomes identified as low or mixed coverage by KvarQ, missing country data, known cross-sectional and longitudinal isolates from the same patient were excluded, and if more than one genome sequence was available for a sample only the first listed was used (*n* = 139 genomes retained). Literature and GMTV database (Chernyaeva et al., 2014) searches, coupled with screening using KvarQ, were also performed to identify additional L4.4 genomes (*n* = 78). BLAST searches identified eight previously sequenced Canadian SUMu strains (Pepperell et al., 2013) belonging to the L4.4 sublineage. Fastq data was unavailable for these genomes therefore new Canadian *Mtb* sequence data was included [23 new and two recently published genomes (Brynildsrud et al., 2018)]. These isolates were selected to encompass a broad range of collection dates and IS*6110*-RFLP molecular typing patterns. Canadian *Mtb* genomes were sequenced using paired-end 250-bp reads on an Illumina HiSeq 2500 using the Nextera^TM^ XT DNA Library Preparation Kit (Illumina Inc., CA, United States) as previously described (Doroshenko et al., 2018). New sequencing data were submitted to the NCBI Nucleotide Archive (PRJNA573497). All 23 new Canadian genomes and 190 publicly available L4.4 genomes passed mapping quality thresholds (depth of coverage > 25X, > 75% reads mapped to the reference genome, and < 10% missing calls in alignments) and were included in downstream analyses.

### Reference Genome Alignment and Variant Calling

Reference genome alignment and variant calling was performed using the Reference Guided Assembly Pepperell Lab Pipeline^1^. Publicly available genomes were downloaded from NCBI using Fastq-dump (v2.5.2). Raw reads were trimmed with TrimGalore! (v0.4.0)^2^ using quality threshold of 15 and reads less than 20 bp long were discarded. Trimmed reads were mapped to the H37Rv reference genome (NC_000962.3) (Cole et al., 1998) using BWA-MEM (v0.7.12) (Li, 2013). Duplicates were removed using Picard tools (v1.138)^3^ and local realignment was performed using GATK (v3.4.46) (DePristo et al., 2011). Mapping quality was assessed using Qualimap (v2.2.1) (Garcia-Alcalde et al., 2012). Genomes were excluded if the depth of coverage was < 25X or if < 75% of trimmed reads mapped to the reference genome. Variants were called using Pilon (v1.16) (Walker et al., 2014) using a minimum depth threshold of 10, base quality threshold of 20 and mapping quality threshold of 40. VCF files generated by Pilon were converted to FASTA format using in house scripts that treat ambiguous calls and deletions as missing data (pilonVCFtoFasta.py).

Bases in repetitive regions of the *Mtb* genome (Supplementary Table S4) were masked using Bedtools (v2.18) (Quinlan and Hall, 2010) and removed from FASTA sequences prior to alignment. Variant sites were extracted from concatenated whole genome alignments using SNP-sites (v2.3.2) (Page et al., 2016). Genomes with missing data at > 10% of sites were excluded from further analyses and only sites where at least 90% of isolates had high quality base calls were included in phylogenetic and molecular dating analyses. VCF and bam files were manually examined for the presence of the DS6^Quebec^ deletion using SAMtools tview (v1.2) (Li et al., 2009) and Artemis (v16.0.0) (Rutherford et al., 2000) (positions 1987457 to 1998849) (Nguyen et al., 2004).

### Maximum Likelihood Phylogenetic Inference

Maximum likelihood trees were inferred using PhyML 3.1 (v3.1) (Guindon and Gascuel, 2003) with x1000 bootstrap replicates using the GTR substitution model as this was the best fitting model based on the Bayesian information criterion in jmodeltest2 (v2) (Darriba et al., 2012). A phylogeny of 23 New Zealand L4.4.1.1 sublineage genomes was inferred from a 345 bp whole-genome SNP alignment. Pairwise SNP distances were calculated from these variant sites using the poppr:bitwise.dist (v2.8.1) package in R (Kamvar et al., 2014) using the “missing_match = T” option to count sites with missing data as matching. A 9024 bp SNP alignment was used to infer a global L4.4 phylogeny, this included all high-quality New Zealand and global L4.4 genomes (*n* = 236, Supplementary Table S2) and H37Rv. The R-package PopGenome (v2.6.1) (Pfeifer et al., 2014) was used to calculate pairwise fixation indices (*F*_*ST*_) from variant sites to estimate population separation between lineages, specifying groups by lineage as determined by KvarQ.

### Bayesian Phylogenetic Analyses

Bayesian evolutionary analysis of the L4.4.1.1 sublineage was performed in BEAST2 (Bouckaert et al., 2014) using 3161 variant sites extracted from a 3949977 bp alignment of 117 L4.4.1.1 genomes with known year of isolation (Supplementary Table S3). XML-input files were manually modified to specify the number of invariant sites as calculated by scaling the number of non-SNP sites in the full alignment by the frequency of each base.

#### Assessment of Temporal Signal for Tip-Based Calibration

The molecular clock was calibrated using tip dates covering a 26-year period (1987–2013). To determine if the temporal signal was sufficient for accurate molecular dating, the dataset was assessed using root-to-tip regression and date randomization (Supplementary Figure S5). A maximum likelihood tree was first constructed in PhyML and then Tempest (v1.5) (Rambaut et al., 2016) was used to root the phylogeny and determine root-to-tip distance for regression analysis against tip date, revealing a modest temporal signal in the data (*R*^2^ = 0.229). The CS clade sample subset (*n* = 47) showed weaker temporal signal than the full L4.4.1.1 dataset (*R*^2^ = 0.139). To further validate the temporal signal, sampling dates were randomized 20 times and analyzed with BEAST2 (v2.4) using a strict clock and constant demographic model with the same parameters for the random and real dates. Estimates of the substitution rate and TMRCA showed no overlap in the 95% HPD between the real and randomized dates, indicating that the data contains sufficient temporal signal for tip-based calibration.

#### Molecular Dating

Mutation rates and divergence times were estimated using MCMC sampling in BEAST2 (v2.4) with the BEAGLE library (Ayres et al., 2012). Analyses were performed using the GTR substitution model, strict and relaxed molecular clocks [uncorrelated relaxed clock with a log-normal distribution (UCLD)] (Drummond et al., 2006), and coalescent constant, exponential and Bayesian skyline (Drummond et al., 2005) demographic models. To correctly place the root as determined with high confidence bootstrap support in the maximum likelihood phylogeny, two monophyletic taxon sets were created to ensure the most deeply rooted L4.4.1.1 clade was placed as an outgroup. Uniform prior distributions were defined for the substitution rate (1 × 10^–10^–1 × 10^–6^ sub/site/yr) and effective population size (upper bound of 1 × 10^10^). For the Bayesian skyline model, the Jeffrey’s (1/X) prior was deselected for the population size parameter as this an improper prior and therefore unsuitable for model evaluation using path sampling. Default priors were used for the remaining parameters. To estimate posterior distributions, three independent chains were run for 100–350 million states sampling every 10000 states. The first 10% of states were discarded as burn-in and chains were assessed for convergence and sufficient mixing (effective sample size > 200 for all parameters) (Supplementary Figure S8). Samples from the three independent chains were combined and parameter estimation based on the combined chain. Median estimates are reported unless otherwise specified. The maximum clade credibility (MCC) tree was estimated from combined tree samples in TreeAnnotator (Supplementary Figures S6, S7).

The performance of various clock and demographic models was evaluated by path sampling analysis in BEAST2 (v2.5) (Lartillot and Philippe, 2006). For each model, 100 path steps were specified using the proportions of a β(0.3, 1.0) distribution and two separate runs were performed per model to check for consistency. The MCMC was also run in the absence of data to sample prior distributions for each model. Comparison of marginal posterior and prior distributions showed a strong signal from the data indicating our results are just not an artifact reflecting the prior (Supplementary Figure S9). The effect of the prior on parameter estimation was also examined by using different upper bounds and the default 1/X prior for the effective population size. Congruent rate and date estimates were obtained when the varying prior parameters on population size demonstrating the robustness of our estimates to this prior specification (Supplementary Figure S10).

#### Phylogeographic Inference

Ancestral reconstruction was performed using BEAST2 (v2.4), with UN region for each isolate modeled as a discrete trait. Analyses were performed using the GTR model of nucleotide substitution, a strict molecular clock with the estimated substitution rate of 6.28 × 10^–8^ sub/site/yr and BSP demographic models. Migration rates over time were inferred from an MCC tree. As described in O’Neill et al. (2019), migration events were defined as a change in the most probable reconstructed state from parent to child node. Only nodes with a posterior probability > 80% were considered. Median heights of the parent and child nodes were treated as the range of time in which a migration event could occur. Migration rates through time were inferred by summing the number of migration events during each year of the phylogeny, divided by the total number of branches in existence during each year of the phylogeny. The Bayesian stochastic search variable selection method (BSSVS) (Lemey et al., 2009) implemented in BEAST2 was used to identify well-supported migration rates between UN regions in the phylogeographic analyses. SpreaD3 (v0.9.7rc) (Vrancken et al., 2016) was used to calculate Bayes factor for each pairwise rate.

## Data Availability Statement

New genome sequencing data generated for this study were deposited in the NCBI BioProject database (IDs: PRJNA356104 and PRJNA573497). Individual accession numbers for genomes analyzed in this study are given in Supplementary Tables S1–S3. The reference guided assembly Pepperell Lab Pipeline is available at GitHub (https://github.com/pepperell-lab/RGAPepPipe).

## Author Contributions

VA, CP, GC, and RC conceived and designed the study. CM performed the phylogenetic and molecular dating analyses and wrote the manuscript. AS performed the phylogeographic inference. DB, SG, and CM compiled the global dataset. NK and SR provided bacterial isolates. HA sequenced isolates. RO’T and SSG provided sequencing data. CM and AS prepared the figures. VA, CP, DB, and SG provided critical comments about the manuscript. All authors reviewed the manuscript.

## Conflict of Interest

The authors declare that the research was conducted in the absence of any commercial or financial relationships that could be construed as a potential conflict of interest.

## Footnotes

1 https://github.com/pepperell-lab/RGAPepPipe

2 https://www.bioinformatics.babraham.ac.uk/projects/trim_galore/

3 https://broadinstitute.github.io/picard/
