# Supplementary material for: Dispersal of Mycobacterium tuberculosis Driven by Historical European Trade in the South Pacific
Source: Front Microbiol. 2019 Dec 4;10:2778. doi: 10.3389/fmicb.2019.02778 (PMC6915100; doi:10.3389/fmicb.2019.02778)
Supplement: Supplementary file 1 [file Data_Sheet_1.pdf]

Supplementary Material

Supplementary Tables

Supplementary Table S1. New Zealand *M. tuberculosis* L4.4 genomes included in this study (excel file).

Supplementary Table S2. *M. tuberculosis* complex L4.4 genomic dataset used for maximum likelihood phylogenetic analysis (excel file).

Supplementary Table S3. *M. tuberculosis* complex L4.4.1.1 genomic dataset used for BEAST2 molecular dating and phylogeographic inference (excel file).

Supplementary Table S4. Repetitive regions of the *M. tuberculosis* genome excluded from phylogenetic analyses (excel file).

Supplementary Table S5. BEAST2 model evaluation by path sampling analysis.

Supplementary Figures

Supplementary Figure S1. Flow chart depicting samples and steps in analyses.

Supplementary Figure S2. Maximum likelihood phylogeny of 23 New Zealand *M. tuberculosis* Rangipo and Otara strain isolates.

Supplementary Figure S3. Global distribution of *M. tuberculosis* isolates included in this study.

Supplementary Figure S4. Maximum likelihood phylogeny of the *M. tuberculosis* complex L4.4 sublineage.

Supplementary Figure S5. Assessment of temporal signal for molecular dating analyses.

Supplementary Figure S6. Dated Bayesian phylogeny of the L4.4.1.1 sublineage showing individual node ages**.**

Supplementary Figure S7. Dated Bayesian phylogeny of the L4.4.1.1 sublineage showing posterior probabilities of individual nodes.

Supplementary Figure S8. Assessment of MCMC chain convergence.

Supplementary Figure S9. Comparison of prior and posterior distributions.

Supplementary Figure S10. Effect of the population size prior on parameter estimation.

Supplementary Table S5. BEAST2 model evaluation by path sampling analysis. Marginal likelihood estimation (MLE) for different clock and population demographic models was determined using path sampling analysis in BEAST2. The GTR nucleotide substitution model was used for all analyses. Mean log-MLEs are reported for two replicate runs performed to check for consistency. Bayes factors calculated relative to the top ranked model.

Rank	Clock model	Demographic model	Log-marginal likelihood	Bayes factor
1	Strict	Skyline	–5342225.5	
2	Relaxed	Skyline	–5342228.9	3.4
3	Relaxed	Constant	–5342259.6	34.1
4	Strict	Constant	–5342261.1	35.6
5	Strict	Exponential	–5342262.4	37.0
6	Relaxed	Exponential	–5342263.2	37.7

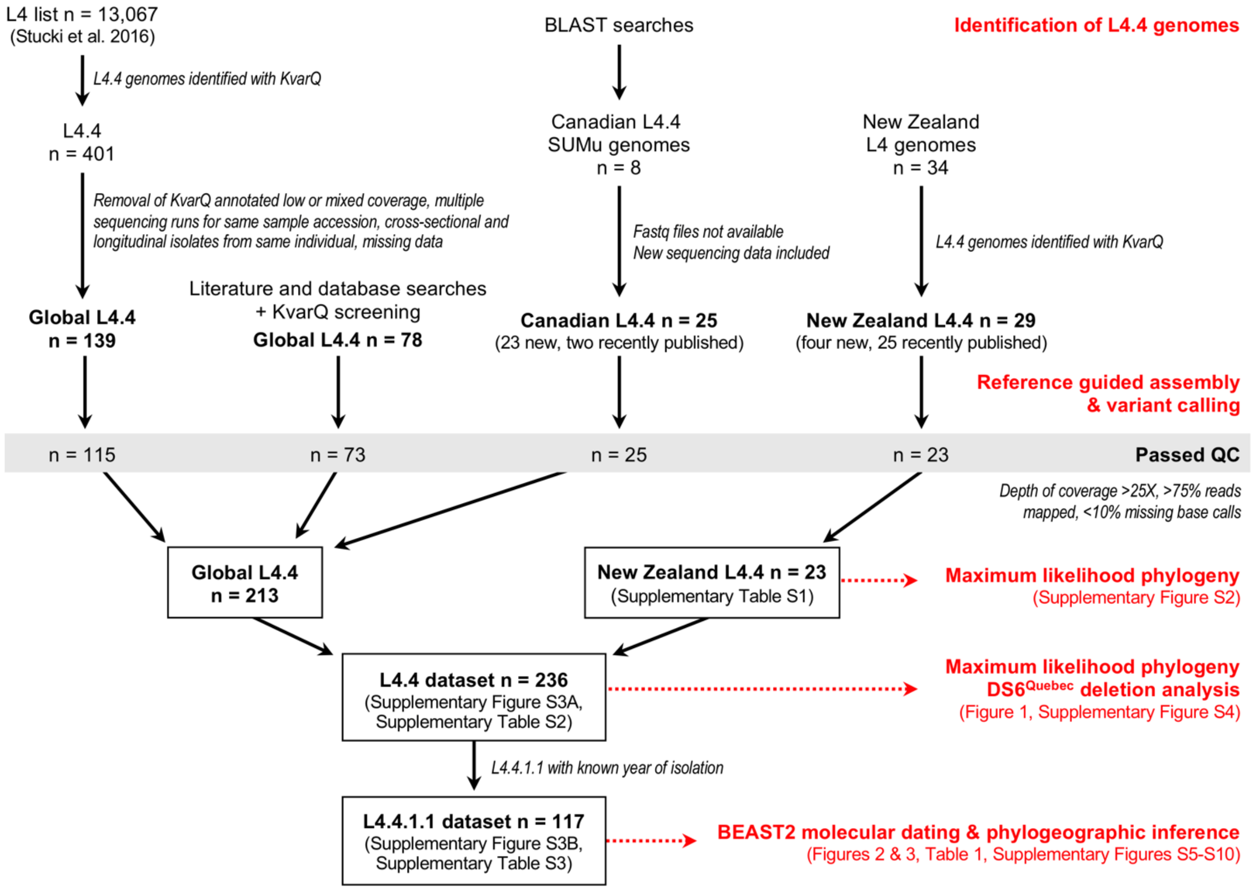

Supplementary Figure S1. Flow chart depicting samples and steps in analyses.

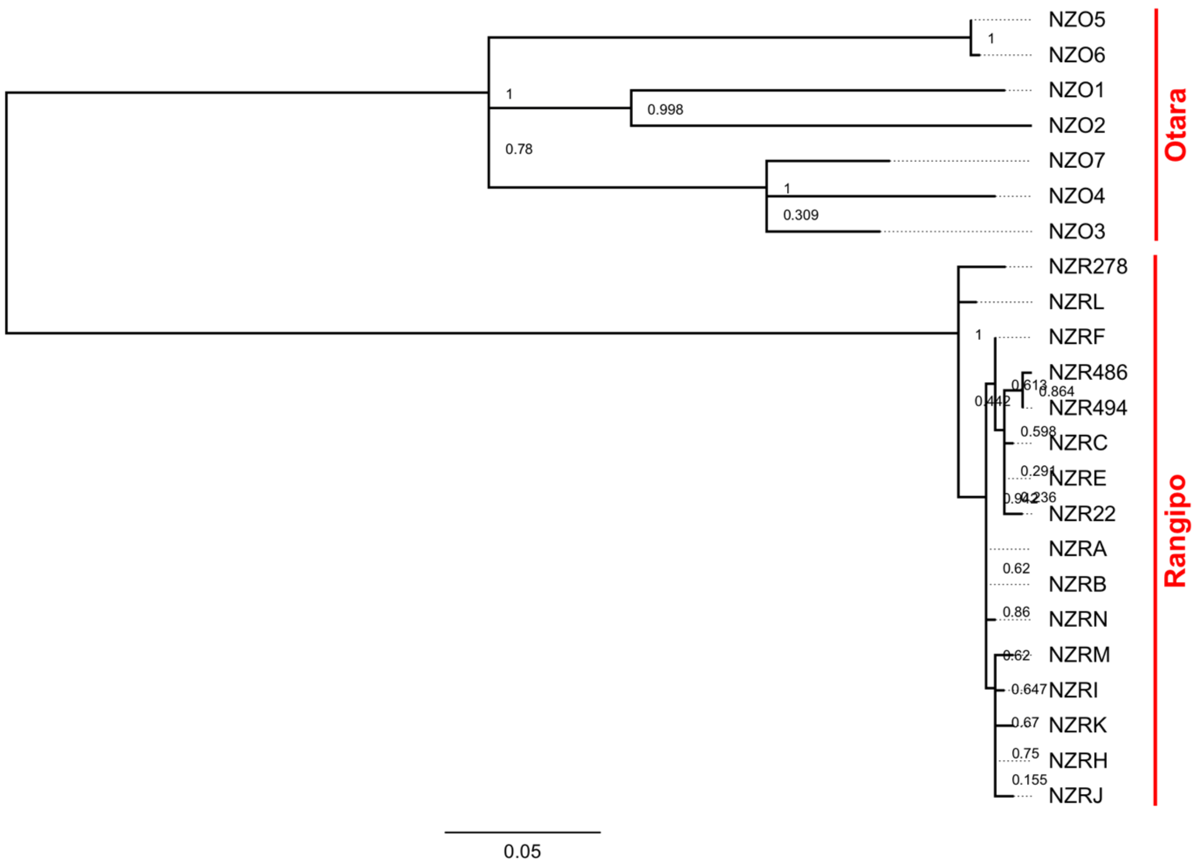

Supplementary Figure S2. Maximum likelihood phylogeny of 23 New Zealand *M. tuberculosis* Rangipo and Otara strain isolates. Scale bar indicates substitutions per polymorphic site.

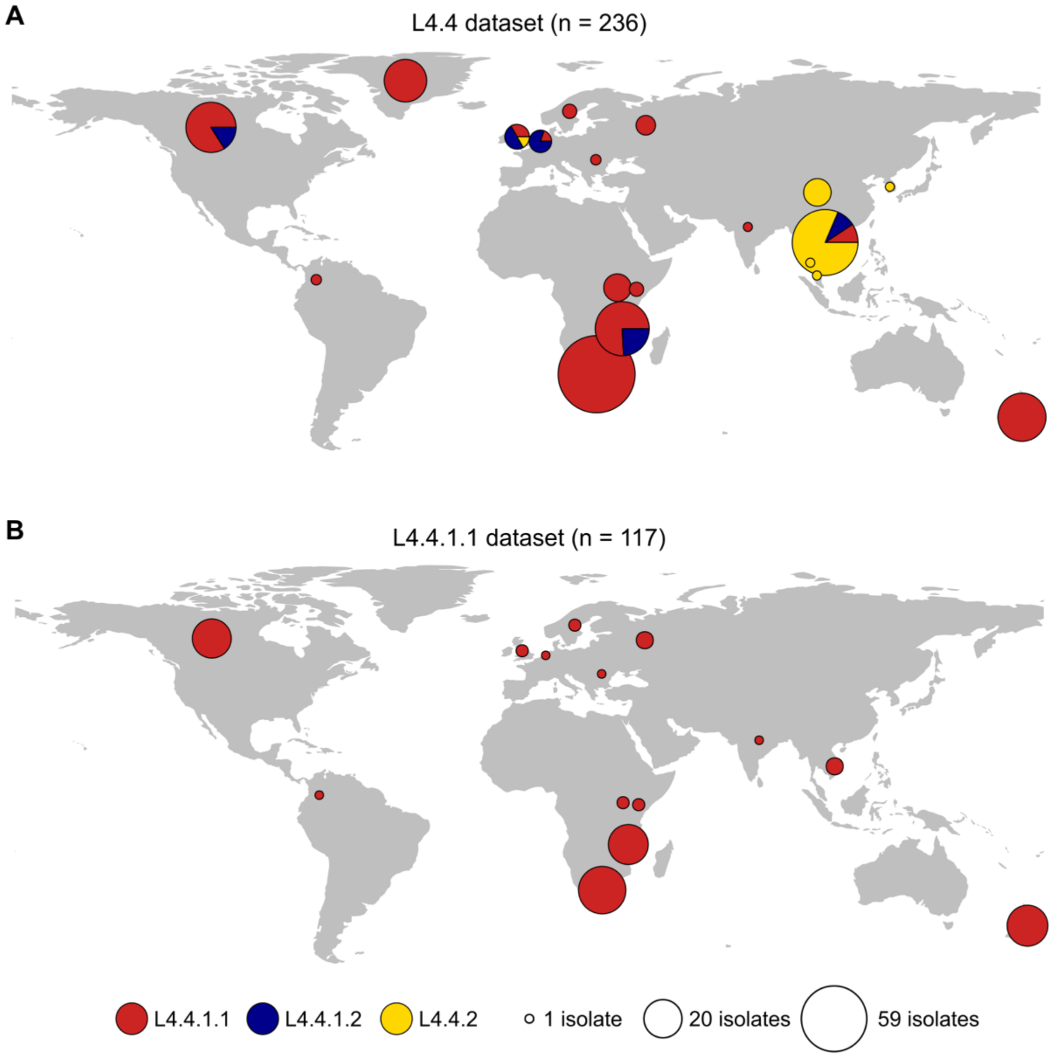

Supplementary Figure S3. Global distribution of *M. tuberculosis* isolates included in this study. (A) L4.4 sublineage genomic dataset used for maximum likelihood phylogenetic analysis; (B) L4.4.1.1 sublineage genomic dataset used for BEAST2 analyses. Pie charts show the proportion of isolates from each of the L4.4 sublineages and circle sizes correspond to the number of isolates from each country.

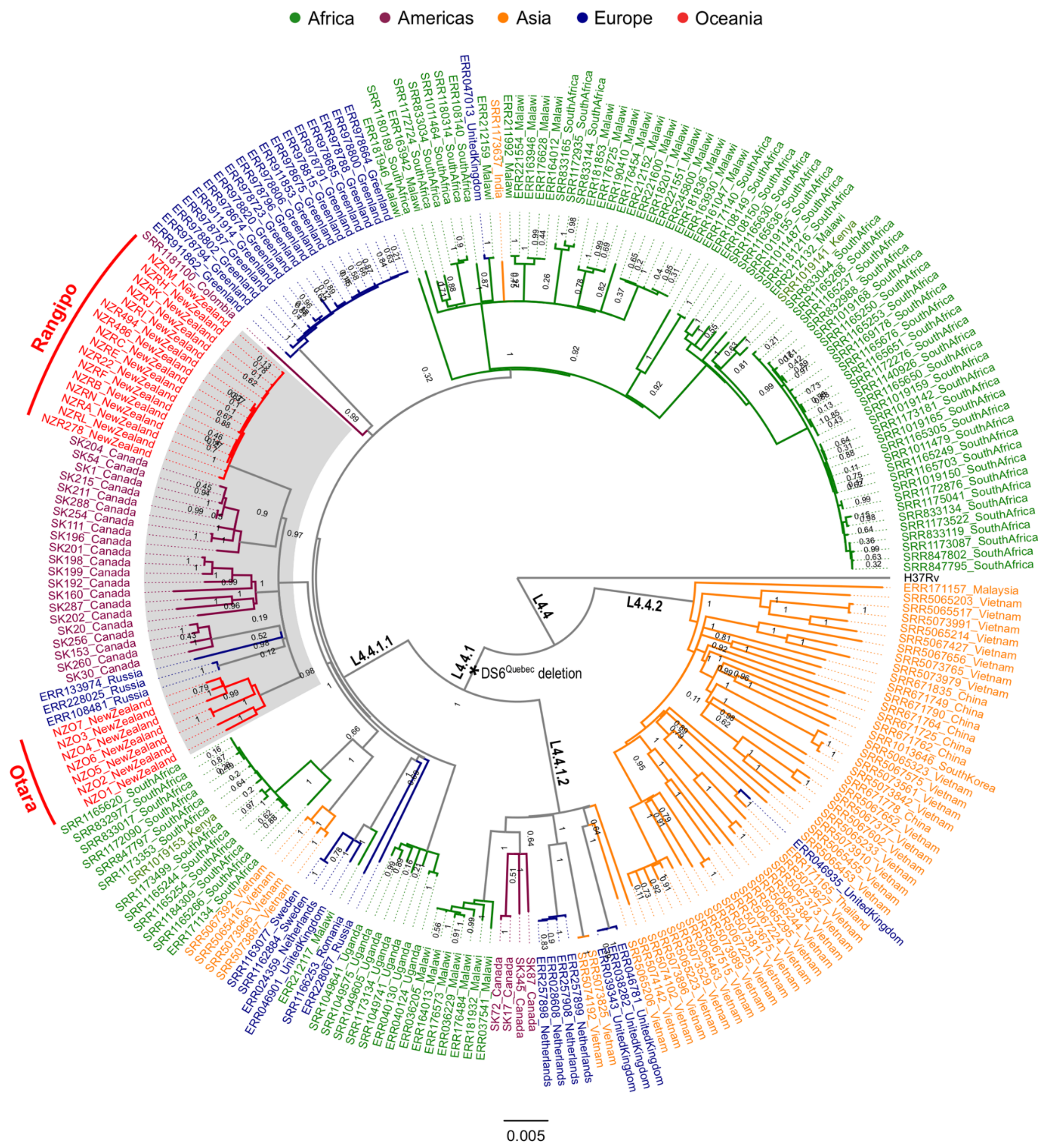

Supplementary Figure S4. Maximum likelihood phylogeny of the *M. tuberculosis* complex L4.4 sublineage. Whole genome SNP phylogeny of 236 L4.4 genomes from 19 different countries, including 23 isolates from the New Zealand Rangipo and Otara clusters. Rooted to H37Rv. Scale bar indicates substitutions per polymorphic site. Branches and tip labels are colored by global region and lineages labelled according to the nomenclature of Coll et al. (2014). A black asterisk indicates the DS6^{Quebec} deletion and the CS clade is highlighted in grey.

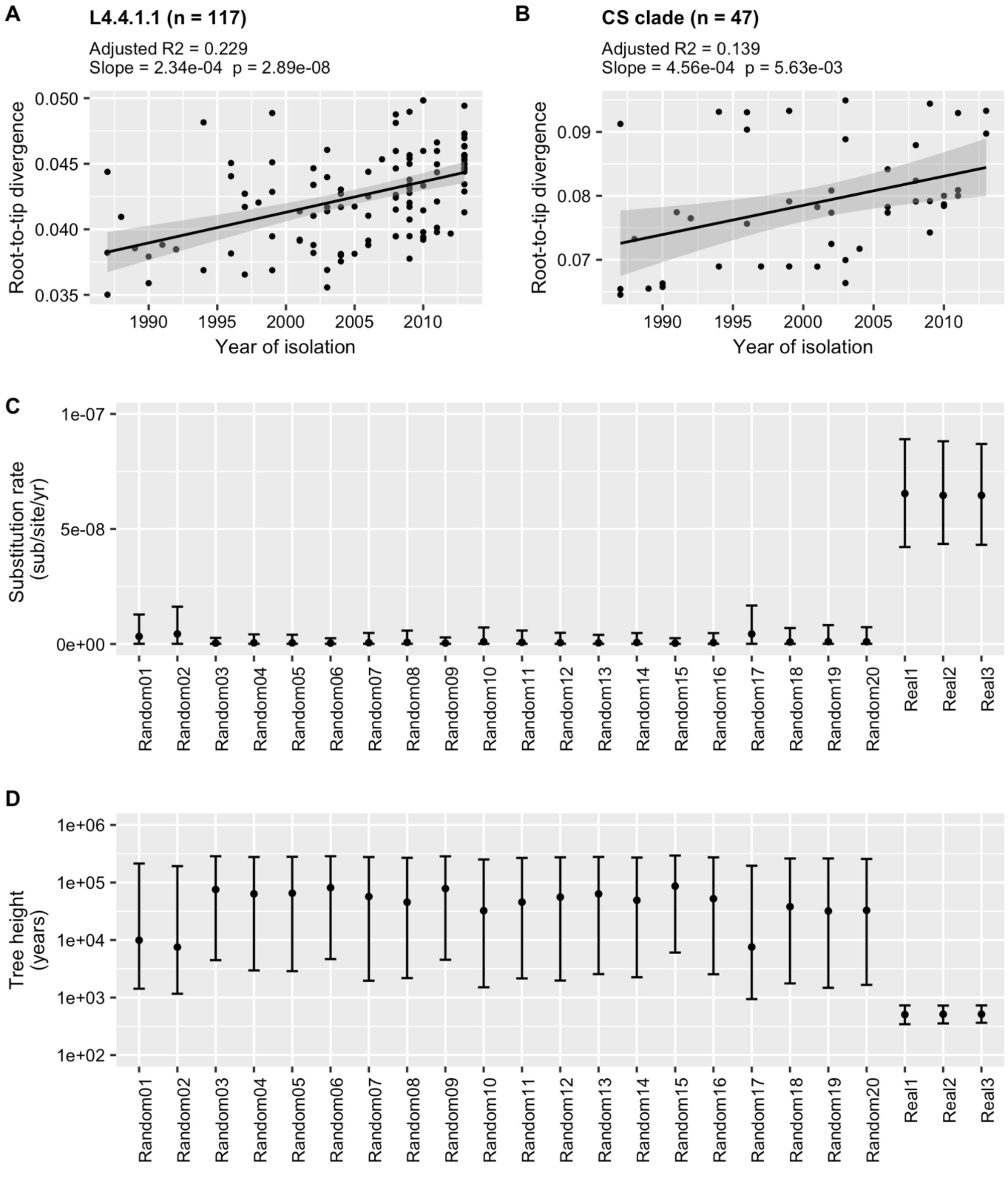

Supplementary Figure S5. Assessment of temporal signal for molecular dating analyses. Regression analysis of root-to-tip distance and year of isolation for (A) the L4.4.1.1 molecular dating dataset, and (B) the CS clade sample subset. Tip date randomization median estimates and 95% HPD intervals for (C) substitution rate in substitutions per site per year (sub/site/yr), and (D) tree height in years, after tip randomization and for real dates for the full L4.4.1.1 sample (n = 117).

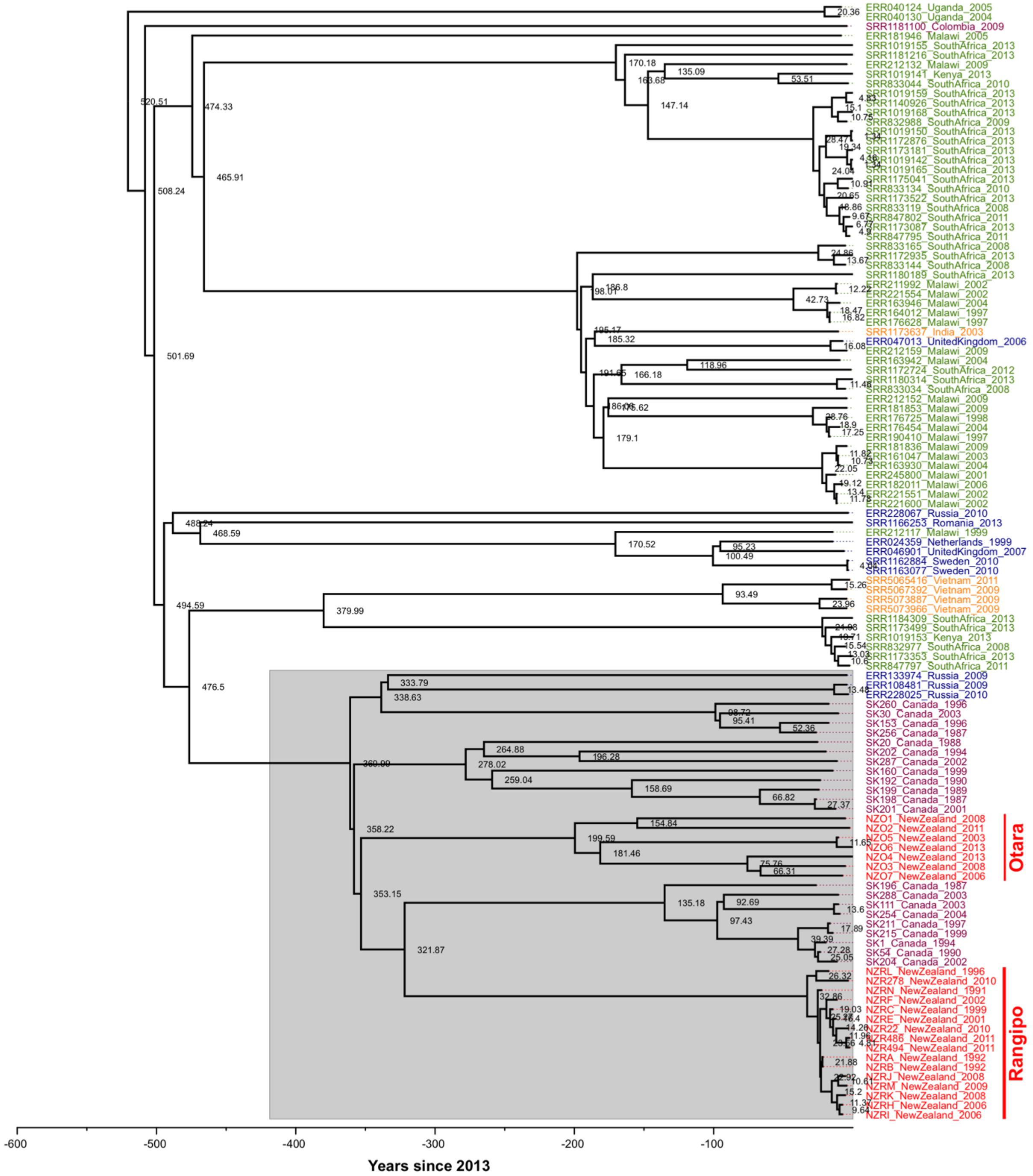

Supplementary Figure S6. Dated Bayesian phylogeny of the L4.4.1.1 sublineage showing individual node ages. Bayesian MCC tree of 117 *M. tuberculosis* L4.4.1.1 genomes (GTR, strict clock, Bayesian skyline demographic). Median node ages (years since 2013) are shown. Genome names are colored by region of isolation. A grey box highlights the CS clade and the New Zealand Rangipo and Otara clusters are labelled.

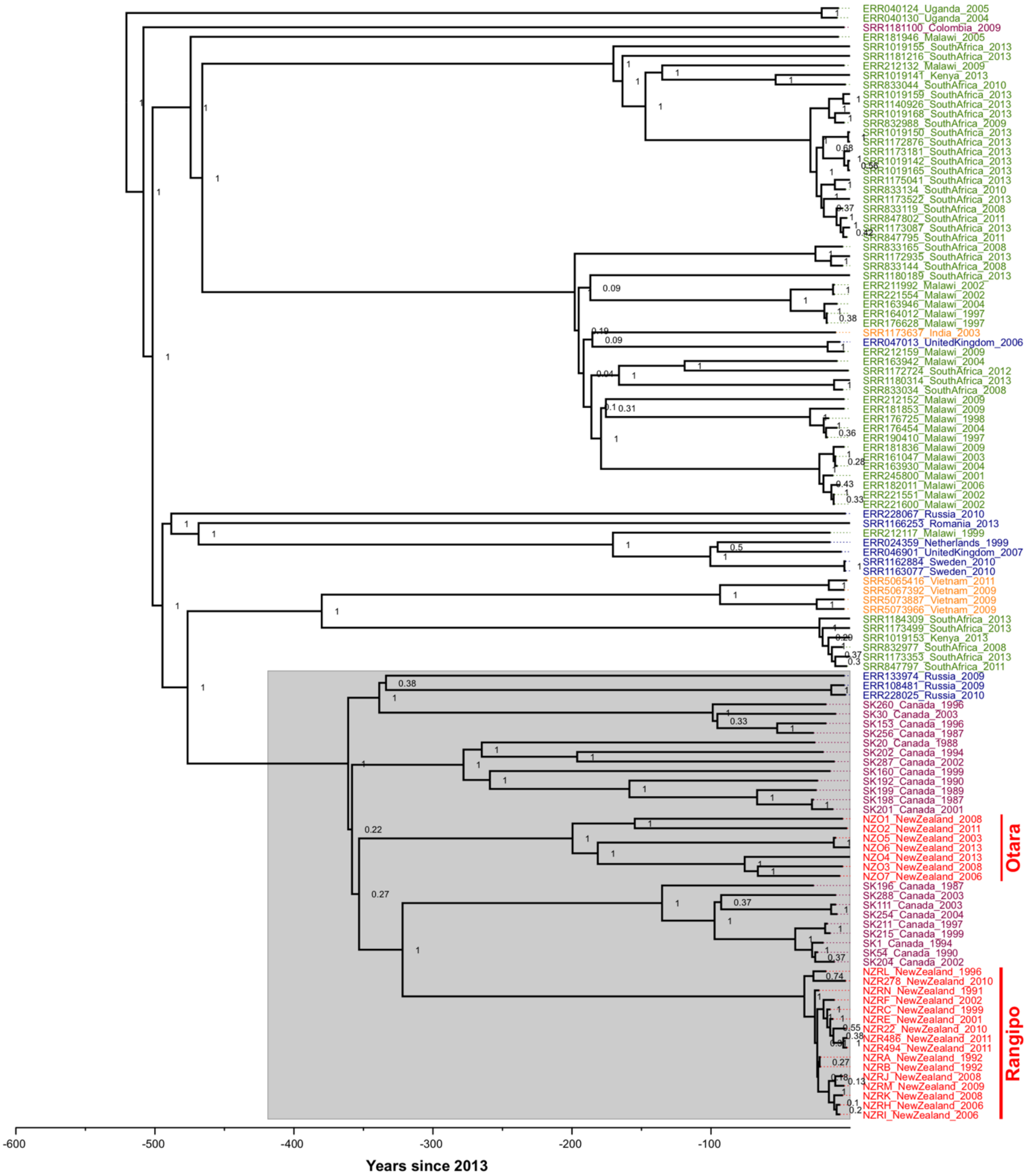

Supplementary Figure S7. Dated Bayesian phylogeny of the L4.4.1.1 sublineage showing posterior probabilities of individual nodes. Bayesian MCC tree of 117 *M. tuberculosis* L4.4.1.1 genomes (GTR, strict clock, Bayesian skyline demographic). Genome names are colored by region of isolation. A grey box highlights the CS clade and the New Zealand Rangipo and Otara clusters are labelled.

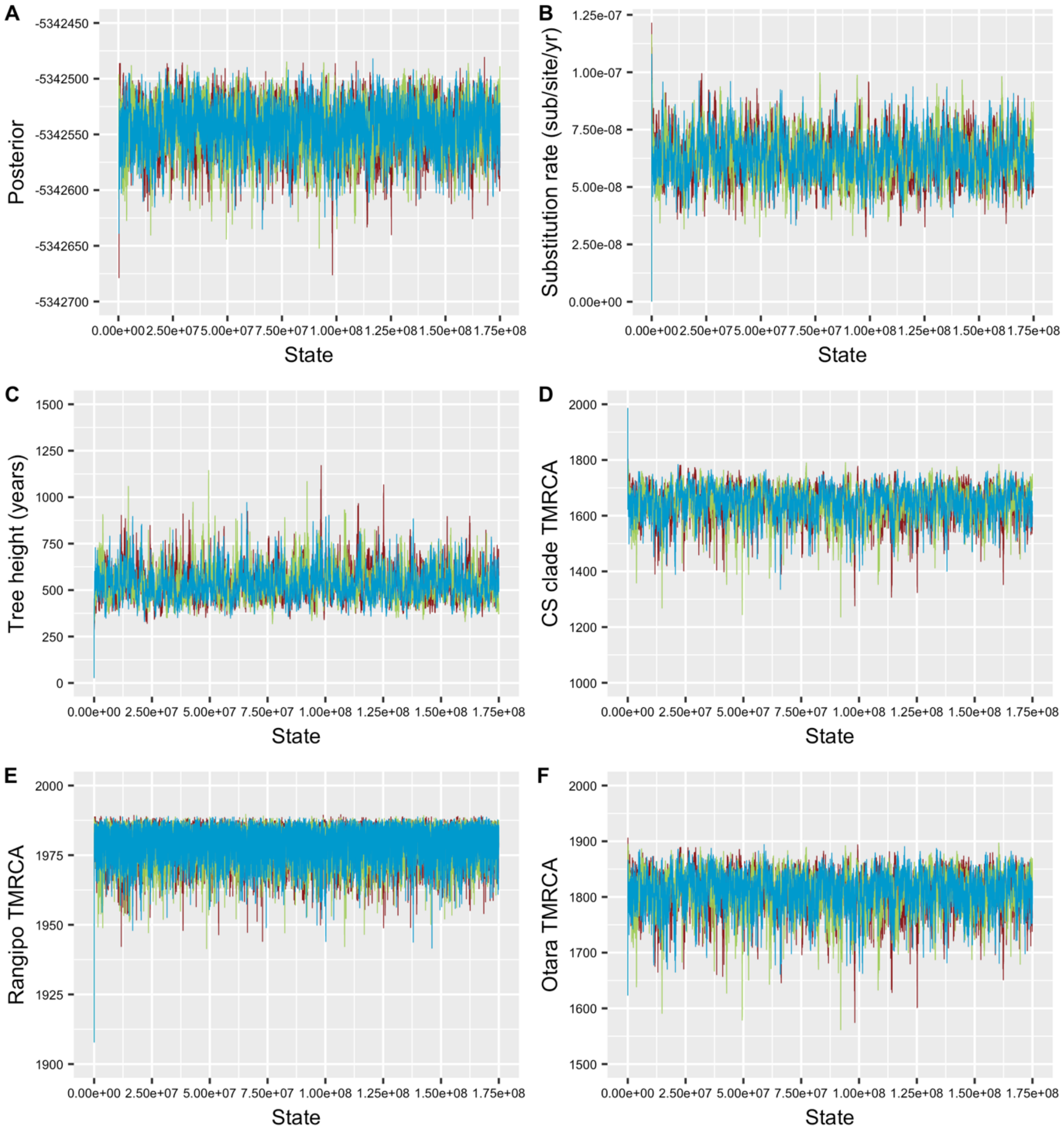

Supplementary Figure S8. Assessment of MCMC chain convergence. Trace outputs for key parameters from three independent chains for the best model as determined by path sampling (GTR, strict clock, Bayesian skyline demographic). (A) Posterior probability; (B) substitution rate in substitutions per site per year (sub/site/yr); (C) tree height; and (D-F) TMRCAs for nodes of key interest in calendar years.

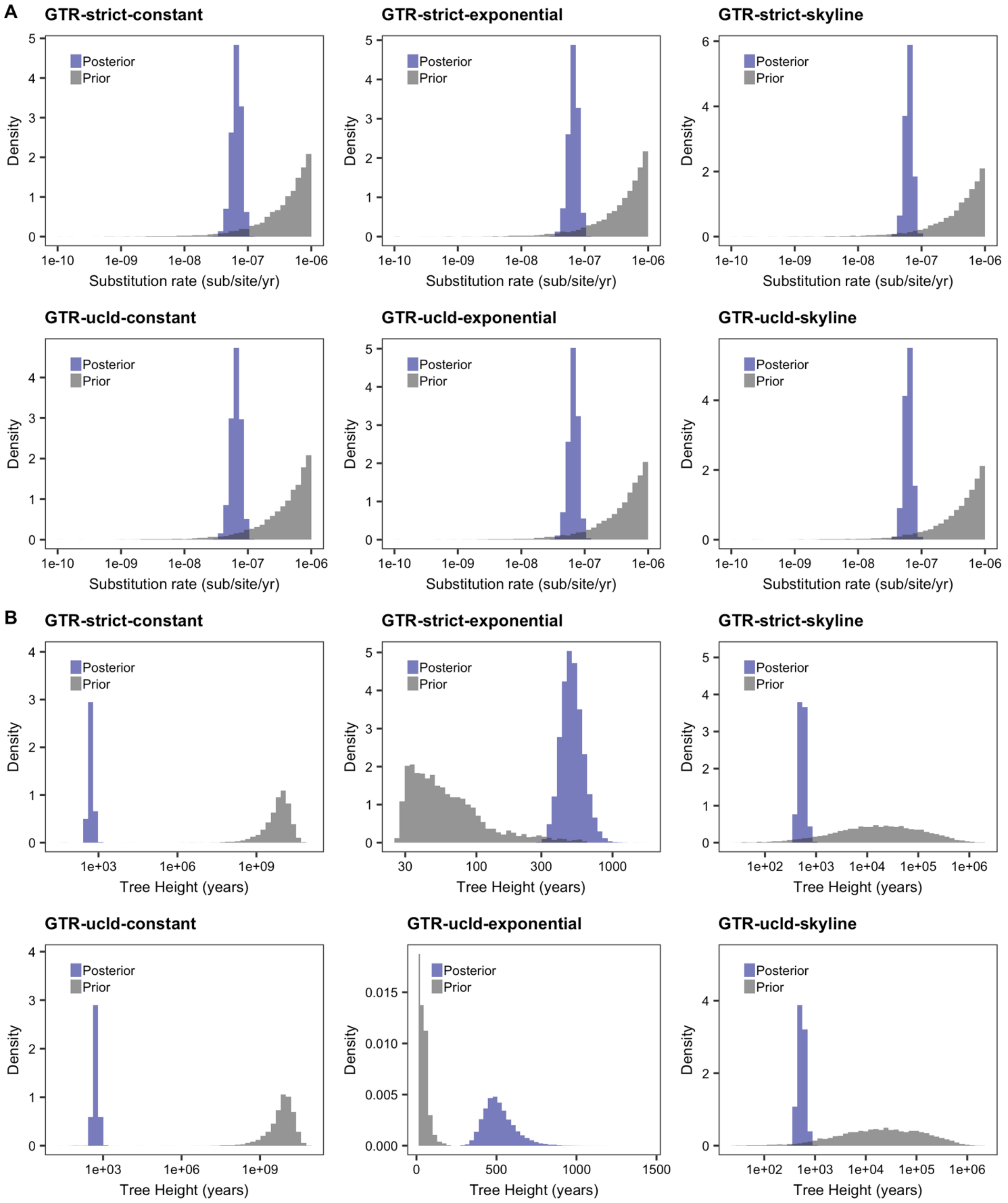

Supplementary Figure S9. Comparison of prior and posterior distributions. (A) Substitution rate in substitutions per site per year (sub/site/yr), and (B) tree height.

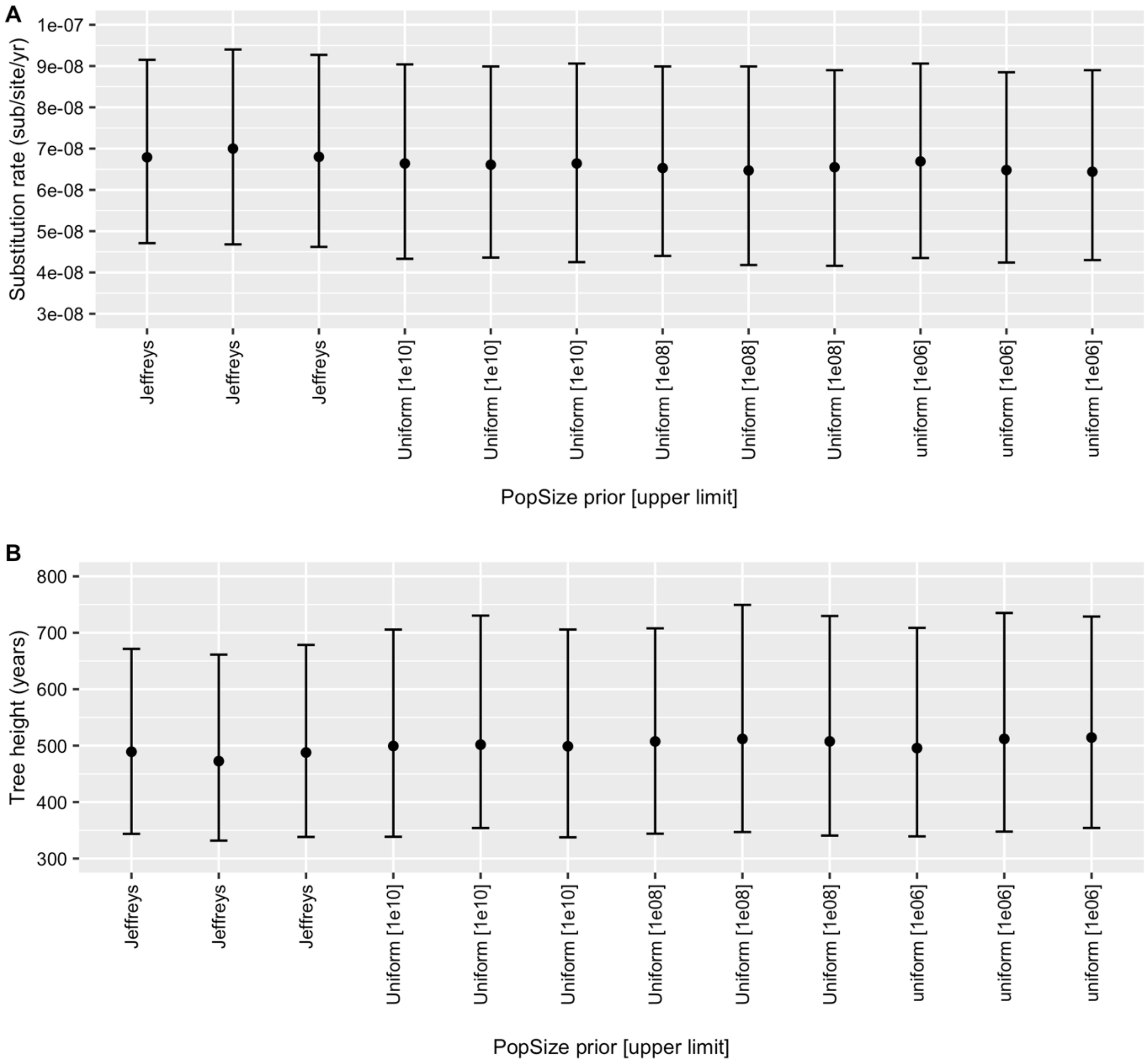

Supplementary Figure S10. Effect of the population size prior on parameter estimation. Comparison of parameter estimates for (A) substitution rate in substitutions per site per year (sub/site/yr), and (B) tree height, using the Jeffreys (1/X) and uniform population size priors with different upper bounds (GTR, strict clock, constant population demographic).
